# A multidomain, nurse-led, mobile health-supported intervention for dementia risk reduction in primary care (HAPPI MIND): a pragmatic, single-blind, two-arm, cluster randomised controlled trial

**DOI:** 10.1016/j.lanwpc.2026.102000

**Published:** 2026-10-20

**Authors:** Muhammad Rehan Sarwar, Kali Godbee, Amanda J. Cross, Gopisankar Mohanannair Geethadevi, Parker Magin, Amanda L. Baker, Billie Bonevski, Sharleen L. O'Reilly, Marlien Varnfield, Rajiv Jayasena, Stephanie A. Ward, Ajay Mahal, Vincent Versace, J. Simon Bell, Kevin Mc Namara, Kaarin J. Anstey, Andrea Hernan, Dennis Thomas, Elizabeth Manias, Rohan A. Elliott, Cik Yin Lee, Mary Tullipan, Denise van den Bosch, Catherine Ferreira, Nina Nguyen, Tesfaye R. Feyissa, Johnson George

**Affiliations:** a Centre for Medicine Use and Safety, Faculty of Pharmacy and Pharmaceutical Sciences, Monash University, Parkville, VIC, 3052, Australia; b School of Medicine and Public Health, The University of Newcastle, Newcastle, NSW, 2308, Australia; c School of Population Health, University of New South Wales, Sydney, NSW, 2052, Australia; d National Drug and Alcohol Research Centre, University of New South Wales, Sydney, NSW, 2031, Australia; e Flinders Health and Medical Research Institute, Flinders University, Bedford Park, SA, 5042, Australia; f School of Agriculture and Food Science, University College Dublin, Dublin, D04 V1W8, Ireland; g School of Exercise and Nutrition Science, Deakin University, Melbourne, VIC, 3125, Australia; h The Australian e-Health Research Centre, Health and Biosecurity, CSIRO, Herston, QLD, 4029, Australia; i The Australian e-Health Research Centre, Health and Biosecurity, CSIRO, Parkville, VIC, 3052, Australia; j Centre for Healthy Brain Ageing, University of New South Wales, Sydney, NSW, 2052, Australia; k School of Public Health and Preventive Medicine, Faculty of Medicine Nursing and Health Sciences, Monash University, St Kilda, VIC, 3004, Australia; l Nossal Institute for Global Health, University of Melbourne, Melbourne, VIC, 3010, Australia; m Deakin Rural Health, School of Medicine, Faculty of Health, Deakin University, Warrnambool, VIC, 3280, Australia; n School of Psychology, University of New South Wales, Sydney, NSW, 2052, Australia; o UNSW Ageing Futures Institute, University of New South Wales, Sydney, NSW, 2052, Australia; p Centre of Excellence in Treatable Traits, College of Health, Medicine and Wellbeing, University of Newcastle, Newcastle, NSW, 2305, Australia; q Asthma and Breathing Research Program, Hunter Medical Research Institute, Newcastle, NSW, 2305, Australia; r Monash Nursing and Midwifery, Faculty of Medicine, Nursing and Health Sciences, Monash University, Clayton, VIC, 3800, Australia; s Pharmacy Department, Austin Health, Heidelberg, VIC, 3084, Australia; t Department of Nursing, School of Health Sciences, Faculty of Medicine, Dentistry and Health Sciences, The University of Melbourne, Melbourne, VIC, 3010, Australia; u North Western Melbourne Primary Health Network, Docklands, VIC, 3008, Australia

**Keywords:** Dementia, Multidomain interventions, Modifiable risk factors, Middle-aged adults, Primary care

## Abstract

**Background:**

Modifiable midlife risk factors contribute substantially to dementia, yet scalable preventive interventions in primary care are limited. We evaluated the effectiveness of a multidomain, nurse-led, mobile health (mHealth)-supported behavioural intervention (HAPPI MIND) among middle-aged adults.

**Methods:**

We conducted a cluster randomised controlled trial in adults aged 45−65 years with ≥2 modifiable dementia risk factors, from 31 Australian general practices (intervention = 13, control = 18). Clinics were randomised to the intervention or control group. Practice nurses collected socio-demographic and clinical data using validated tools. Participants completed the Australian National University–Alzheimer's Disease Risk Index (ANU-ADRI) dementia risk assessment, which assessed exposure to modifiable risks and protective factors for dementia. Intervention participants received five nurse-led motivational interviewing sessions over 12 months, an individualised action plan, and access to the mHealth application. Control participants received the ANU-ADRI report, an educational booklet on dementia risk reduction, and referral to their general practitioner as appropriate. Outcome assessors and data analysts were blinded. The primary outcome was the change in ANU-ADRI score from baseline to 12 months. Analyses followed intention-to-treat principles using linear mixed-effects models with multiple imputation for missing data. The trial was prospectively registered (ACTRN12621001168842); the intervention is complete, and this report presents the prespecified primary outcome at 12 months.

**Findings:**

Between February 2022 and April 2023, 403 participants (intervention = 201; control = 202) were enrolled (mean age 56.4 years [SD 5.7]; 63% female). At 12 months, the adjusted mean change in ANU-ADRI score was 0.791 (95% CI −0.895 to 2.478) in the intervention group and −0.807 (−2.339 to 0.725) in the control group, with an adjusted between-group mean difference of 1.598 (0.423–2.773; p = 0.0080), favouring the intervention. Results from per-protocol and complete-case analyses were consistent. Seven adverse events occurred, including two deaths (one in each group).

**Interpretation:**

A multidomain, nurse-led, mHealth-supported behavioural intervention produced modest but statistically significant improvements in estimated dementia risk profiles over 12 months among middle-aged adults in Australian primary care. Further implementation, processes, and economic evaluations are needed before broader adoption can be recommended.

**Funding:**

Australian Government National Health and Medical Research Council, Boosting Dementia Research Grants (GNT1171851).

**Research in context**

**Evidence before this study**

We searched PubMed and Embase from inception to date, using combinations of terms related to *“dementia prevention”*, *“cognitive decline”*, *“multidomain intervention”*, *“primary care”*, *“nurse-led”*, *“digital health”*, *“mobile health”*, and *“motivational interviewing.”* No language restrictions were applied. Reference lists of relevant systematic reviews and trials were also screened. The relevance and methodological characteristics of the identified studies were considered when interpreting the available evidence; a formal systematic risk-of-bias assessment was not undertaken for this contextual literature search. Previous landmark multidomain prevention trials — such as FINGER, MAPT, and PreDIVA — demonstrated that combined lifestyle interventions targeting vascular and behavioural risk factors can slow cognitive decline in older adults at increased risk of dementia. However, these studies were conducted in more structured research settings than routine primary care practice, required intensive supervision and resources, and mainly involved participants aged ≥60 years. Recent trials, including PRODEMOS, have incorporated pragmatic primary care delivery and digital health approaches to support dementia-risk reduction. To our knowledge, no published randomised controlled trials before HAPPI MIND evaluated a nurse-led multidomain mHealth intervention targeting midlife adults (aged 45–65 years) that was specifically designed for implementation within existing general practice structures in Australia.

**Added value of this study**

HAPPI MIND is the first Australian primary care trial to evaluate a practice nurse-led, multidomain behavioural intervention for dementia risk reduction, supported by mHealth. The intervention combined personalised dementia-risk feedback, targeted behavioural counselling using motivational interviewing, and digital self-monitoring over 12 months. The study demonstrated modest improvements in overall dementia-risk profiles compared with controls, with the greatest improvements observed among participants whose modifiable risk factors were actively targeted (e.g., hypertension, overweight/obesity, low cognitive activity, social isolation, and depression). These findings suggest that personalised nurse-led behavioural support, complemented by digital tools, can achieve modest improvements in estimated dementia-risk profiles within Australian primary care. HAPPI MIND used existing primary care infrastructure, trained practice nurses, and digital support, demonstrating the potential to deliver this model within routine health systems, although formal implementation and economic evaluations are necessary before broader adoption.

**Implications of all the available evidence**

Collectively, available evidence supports multidomain lifestyle interventions for cognitively healthy or at-risk adults as a promising strategy for improving dementia-risk profiles, particularly when delivered early in midlife. HAPPI MIND extends this evidence by demonstrating that a nurse-led, mHealth-supported behavioural intervention can achieve modest improvements in estimated dementia-risk profiles at 12 months within Australian primary care. Embedding nurse-led dementia-risk assessment and behavioural support, drawing on motivational interviewing and cognitive-behavioural therapy principles, within existing chronic disease prevention and management frameworks represents a promising approach. The flexibility to deliver individualised sessions face-to-face, by telephone, or via video telehealth may also facilitate implementation within routine primary care.

However, these findings should be interpreted in the context of the modest effect size, the absence of significant between-group differences in objective clinical outcomes, and the relatively short follow-up period. Further evaluation through longer-term implementation, process, and economic studies is warranted before broader adoption can be recommended. Integration with digital tools may facilitate ongoing engagement and self-management, although engagement with the mHealth component was variable and the independent contribution of the digital component to the observed intervention effects could not be determined. Future research should assess longer-term cognitive and clinical outcomes, implementation strategies, and intervention sustainability across diverse and underserved populations, including rural and lower socioeconomic groups. Together, the current evidence suggests that nurse-led, multidomain, digitally supported prevention models have the potential to improve estimated dementia-risk profiles within primary care; however, the sustainability of these improvements and their potential to translate into reductions in dementia incidence or other clinically meaningful outcomes remains to be established.

## Introduction

The burden of dementia is increasing rapidly due to population ageing,^1^ yet effective disease-modifying or curative treatments remain limited. Accordingly, attention has focused on prevention through modification of lifestyle and vascular risk factors, particularly during midlife — a critical window when intervention may confer the greatest long-term benefit.^2^

Multidomain prevention trials, such as FINGER and US POINTER have demonstrated that targeting several modifiable risk factors simultaneously can reduce cognitive decline and improve estimated dementia risk profiles.^3^, ^4^ Digital and hybrid delivery models such as the PRODEMOS and Maintain Your Brain trials and other mobile health (mHealth)-supported interventions, have further shown promise in improving dementia-risk scores and expanding access.^5^, ^6^ Although several multidomain interventions have incorporated real-world implementation aspects, including primary care and digitally supported delivery, evidence remains limited for pragmatic, practice nurse-led dementia risk reduction programmes integrated within routine Australian primary care and targeting middle-aged adults. Translating these advances into scalable, sustainable, and equitable healthcare models remains an ongoing challenge.

Primary care offers a promising platform for dementia prevention, given its accessibility and central role in chronic-disease management and health-behaviour modification. However, most dementia-prevention trials have not fully leveraged the primary care workforce, particularly practice nurses, who are ideally positioned to deliver tailored behavioural interventions at scale.^7^ As with many countries, Australian general practice operates within a predominantly publicly funded primary care system in which practice nurses play an increasingly important role in chronic disease prevention and management. Nurses represent the largest global healthcare workforce and there is increasing international emphasis on expanding nursing roles to improve access to timely, preventive, and person-centred care, particularly within primary care and underserved settings. Practice nurses are routinely involved in chronic disease prevention, provide lifestyle counselling, and maintain ongoing patient relationships. In addition, increasing workload and time constraints within general practice may limit the capacity of general practitioners to deliver repeated behavioural counselling, whereas practice nurses are well positioned to provide ongoing, personalised support and behavioural-change interventions. Their accessibility, continuity of care, and training in patient education and motivational approaches may facilitate sustained engagement and implementation of multidomain dementia prevention strategies within routine primary care. Integrating nurse-led care with mHealth applications may enhance patient engagement, support sustained self-management and extend behaviour-change beyond clinical consultations. The Holistic Approach in Primary care for PreventIng Memory Impairment aNd Dementia (HAPPI MIND) trial was designed to evaluate the effectiveness of a practice nurse-led, mHealth-supported, multidomain intervention to improve estimated dementia risk profiles in middle-aged adults within primary care, while also exploring implementation considerations relevant to feasibility and scalability within routine general practice settings.^8^ We aimed to determine whether the HAPPI MIND intervention improved estimated dementia risk profiles, as measured by the Australian National University Alzheimer's Disease Risk Index (ANU-ADRI), over 12 months, compared with a control intervention, and to assess harms during the 12-month follow-up. We hypothesised that the intervention would improve estimated dementia-risk profiles and selected health-related behavioural outcomes over 12 months compared with usual care.

## Methods

### Study design

A pragmatic, open-label, single-blind, two-arm cluster randomised controlled trial was conducted in general practice clinics across Victoria and New South Wales, Australia. Clusters were defined at the practice level to minimise contamination between intervention and control participants. An open-label design was selected because the intervention's behavioural and mHealth components made participant and provider blinding unfeasible. Partial disclosure of specific study objectives was implemented, whereby participants were informed that the study aimed to assess general health and lifestyle factors, without emphasising dementia risk reduction as the primary outcome. This approach was intended to minimise expectation bias and potential Hawthorne effects; although repeated self-reported assessments may still have influenced behavioural awareness and reporting over time.

The trial was conducted and reported in accordance with the Consolidated Standards of Reporting Trials (CONSORT) 2025 statement and the CONSORT extension for cluster randomised trials.^9^ A study protocol was published previously,^8^ and no major amendments were made after the trial commenced.

The study was approved by the Monash University Human Research Ethics Committee (Reference: 28273) and conducted in accordance with the ethical standards as laid down in the 1964 Declaration of Helsinki and its later amendments or comparable ethical standards.

Clinics were randomised before participant recruitment. Potential participants were informed that their clinic had been allocated to one of two health-promotion programmes but were not informed of their clinic's specific treatment allocation before providing written informed consent. To minimise expectation bias, the specific hypothesis regarding dementia risk reduction was not emphasised during recruitment (partial disclosure). The trial was prospectively registered with the Australian and New Zealand Clinical Trials Registry (ACTRN12621001168842) and is closed to new participants. The intervention comprised six practice nurse-led sessions, with five delivered during the first 12 months and a sixth booster session at 24 months. Follow-up to 36 months will evaluate the sustainability of intervention effects and longer-term outcomes. The primary outcome is change in ANU-ADRI score at 12 months.

Consumer perspectives informed the development of the HAPPI MIND intervention. During the qualitative phase preceding the trial, consumers were interviewed, and their perspectives were incorporated into the design of the intervention.

### Participants

Group or solo general practice clinics in metropolitan or regional areas of Victoria and New South Wales with at least 500 middle-aged (45–65 years) active patients (defined as ≥1 visit in the past 12 months) were approached directly or through primary health networks. Practices were required to provide a private consultation area with internet access, a nurse willing to complete study-specific training, and capacity for intervention delivery, or to accommodate an external intervention nurse.

Eligible participants were community-dwelling adults aged 45–65 years who attended the practice within the previous 12 months, had two or more modifiable dementia risk factors (e.g., hypertension, obesity, physical inactivity; risk factors definitions and assessment methods are provided in Supplementary Table S1), and were able to use a smartphone or tablet. Potential participants were identified through electronic medical record searches and opportunistic recruitment during clinic visits.

Participants were excluded if they were unable to provide informed consent or communicate in English, had a terminal illness (anticipated survival <36 months), a diagnosis of dementia, mild cognitive impairment, or other significant neurological disease, were currently enrolled in another clinical trial targeting modifiable dementia risk factors, or had other conditions deemed by the general practitioner to limit participation.

Each clinic aimed to recruit an average of 15 eligible participants, with the option to recruit up to 30 participants if capacity permitted, until the overall trial recruitment target was reached. Sex and gender were self-reported. Detailed race and ethnicity data were not collected because these variables were not included in the original trial data-collection framework; however, Indigenous status, country of birth, and language spoken at home were recorded.

### Randomisation and masking

After obtaining written agreement, general practice clinics were randomised at the cluster level using computer-generated block randomisation (block size = 4) with a 1:1 allocation ratio. The randomisation sequence was generated in Stata (version 16; StataCorp, College Station, TX, USA) and prepared by an independent statistician not involved in recruitment or analysis. Allocation was concealed until clinics were notified of their group allocation assignment.

Trained practice nurses and research assistants identified potentially eligible clinic participants through clinical database searches and subsequent telephone, text message, email, or post contact.

Blinding of staff delivering the intervention and participants was not feasible; however, outcome assessors and data analysts remained blinded to group allocation throughout data collection and analysis. The primary outcome (ANU-ADRI) was completed by participants either independently through a secure online questionnaire or administered by trained research assistants blinded to treatment allocation, who recorded participants’ responses using standardised procedures. The mode of administration was determined by participant preference and practical considerations (e.g., location and availability), and the same mode was used across study visits whenever feasible. In both modes, ANU-ADRI responses were provided by participants rather than assessed or rated by research staff. Practice nurses delivering the intervention were not involved in follow-up outcome assessment procedures.

### Procedures

The intervention comprised a practice nurse-led, multidomain dementia risk-reduction program delivered within primary care (Supplementary Figure S1). Practice nurses completed a two-day training program led by a psychologist, covering motivational interviewing and cognitive-behavioural therapy principles, followed by ongoing supervision during the first three months of implementation.

Using behaviour-change techniques, nurses supported participants to identify and manage modifiable dementia risk factors across vascular (e.g., hypertension, diabetes, dyslipidaemia), lifestyle (e.g., physical activity, diet, alcohol use, smoking), and psychosocial domains (e.g., social engagement, cognitive stimulation, mood). Cognitive activity was addressed through general cognitive stimulation strategies, such as reading, puzzles, learning activities, and social engagement, rather than structured cognitive training programs. Nurses were trained to provide balanced risk communication and avoid framing dementia as entirely preventable through lifestyle modification alone. Participants were informed during the consent process and behavioural coaching sessions that modifiable dementia risk factors influence dementia risk at a population level, but do not guarantee prevention of dementia at an individual level. Each participant received an individualised dementia risk report derived from their baseline ANU-ADRI assessment. The report identified 14 modifiable dementia risk factors targeted through personalised behavioural counselling and goal-setting strategies. Participants developed a personalised action plan in collaboration with the practice nurse, informed by the COM-B (capability, opportunity, motivation-behaviour) framework^10^ and SMART (specific, measurable, attainable, realistic, timely) goal-setting principles.^11^ Goals could include behavioural targets (e.g., physical activity, diet, cognitive engagement, smoking cessation) and vascular risk factors management in collaboration with participants’ general practitioners.

Intervention participants had access to the HAPPI MIND mHealth application (developed by the Commonwealth Scientific and Industrial Research Organisation), which enabled self-monitoring of lifestyle behaviours, goal tracking, and access to educational content and reminders, while allowing nurses to review progress via a clinician web dashboard. The intervention was delivered through five motivational interviewing sessions over 12 months, which could be conducted face-to-face, by telephone, or via video consultation according to participant and practice circumstances, supplemented by ongoing app-based engagement and feedback. At each session, practice nurses reviewed participants' progress towards previously agreed goals, reassessed relevant modifiable dementia risk factors, reinforced behaviour-change strategies, and updated personalised action plans where appropriate. The intervention manual provided practical guidance on assessing each risk factor, supporting behaviour change, monitoring progress using the HAPPI MIND app, and identifying when participants should be referred to their general practitioner for further assessment, treatment, or referral to allied health or specialist services. Nurses also encouraged participants to engage in cognitive, social, and physical activities consistent with their personalised goals and preferences.

Control participants received a brief intervention at baseline, based on the screening, brief intervention, and referral to treatment (SBIRT) framework.^12^ Trained nurses provided a one-off session that included screening for modifiable dementia risk factors using the ANU-ADRI questionnaire, generation of an individualised dementia risk report, and provision of an educational booklet on dementia risk reduction identical to that provided to intervention participants. Following the session, participants were advised to consult their general practitioner to discuss identified risk factors, potential need for lifestyle modification, or referral to allied health or specialist services. Control participants continued to receive usual care from their primary care practice, including routine management of vascular and lifestyle risk factors, but without the structured nurse-led intervention or mHealth support.

Following written informed consent, all baseline assessments, including the ANU-ADRI questionnaire and other self-reported outcome measures, were completed prior to any intervention components delivery. Baseline demographic and clinical data were collected by trained practice nurses at baseline and 12 months post-randomisation, using a combination of clinical assessments, validated questionnaires, and digital application logs. Trained assessors blinded to group allocation performed all cognitive and clinical assessments. Up to ten contact attempts were made to minimise loss to follow-up. Data were entered and managed using the REDCap electronic data-capture tool hosted and managed by Helix (Monash University), with inbuilt range and logic checks to promote data accuracy.

Participant socio-demographic characteristics were collected during interviews, and medical records were reviewed to obtain medical history. In addition, participants were assessed at baseline and 12-months follow-up using validated tools including, ANU-ADRI,^13^ cardiovascular risk factors ageing and dementia risk score (CAIDE),^14^ montreal cognitive assessment (MoCA),^15^ international physical activity questionnaire (IPAQ) short form,^16^ centre for epidemiologic studies depression (CES-D) scale,^17^ EQ-5D-5L,^18^ mediterranean-dietary approaches to stop hypertension (DASH) diet intervention for neurodegenerative delay (MIND) questionnaire,^19^ Australian type 2 diabetes risk assessment tool (AUSDRISK),^20^ absolute cardiovascular disease (CVD) risk,^21^ and alcohol use disorders identification test (AUDIT).^22^

Clinical parameters (height, weight, blood pressure, total cholesterol, high-density lipoprotein cholesterol [HDL-C], low-density lipoprotein cholesterol, non-HDL-C, triglycerides, blood glucose, and glycated haemoglobin A1c) were also collected from participants at baseline and 12 months follow-up.

### Outcomes

The primary outcome was the mean change in estimated dementia risk, measured by the ANU-ADRI score, from baseline to 12 months. In addition, exploratory analyses examined changes in the ANU-ADRI risk factor and protective factor sub-scores to provide further insight into specific behavioural and vascular domains contributing to overall risk reduction.

The ANU-ADRI is a validated self-report risk index that estimates future dementia risk based on modifiable and non-modifiable factors. The individual ANU-ADRI components, scoring approach, and classification as risk, protective, or mixed domains are summarised in Supplementary File 2. Higher scores indicate greater risk, while score reductions over time reflect a more favourable dementia risk profile. Although originally developed for Alzheimer's disease risk prediction, it has since been expanded to reflect broader dementia risk.^13^, ^23^ It was selected as the primary outcome due to its ability to capture multidomain dementia risk factors and its suitability for evaluating behavioural interventions in population-based settings.^6^, ^24^, ^25^ ANU-ADRI has previously been used in multidomain dementia-prevention trials to evaluate changes in estimated dementia-risk profiles over time. It was selected as the primary outcome because it captures changes across multiple modifiable risk and protective factors targeted by the intervention and is therefore aligned with the behavioural risk-reduction focus of HAPPI MIND. Cognitive outcomes were assessed separately as secondary outcomes.

Secondary outcomes included change in dementia risk using clinical parameters (CAIDE score; a complementary clinically derived risk measure incorporating objective cardiometabolic factors to allow comparison with the primarily self-reported ANU-ADRI); change in cognitive performance (MoCA); change in health-related quality of life (EQ-5D-5L); and change in cardiovascular risk. Additional secondary outcomes included changes in psychosocial and behavioural measures from baseline to 12 months, assessed using the CES-D (depressive symptoms), MIND diet (dietary quality), AUDIT (alcohol use), AUSDRISK (diabetes risk), patient-reported outcomes measurement information system satisfaction with participation in discretionary social activities (PROMIS—social participation), patient activation measure (PAM—self-management activation), tool for adherence behaviour screening (TABS—medication adherence behaviours), self-administered comorbidity questionnaire (SCQ—comorbidity burden), STOP-BANG questionnaire (snoring, tiredness, observed apnoea, high blood pressure, body mass index, age, neck circumference, and gender—sleep apnoea risk), and insomnia severity index (ISI—insomnia symptoms).

For participants in the HAPPI MIND intervention group, process measures included the number of motivational interviewing sessions attended and engagement with the HAPPI MIND mobile application, measured by frequency of app use and data entry.

Adverse events and serious adverse events were systematically monitored throughout follow-up. Events reported by participants or identified by practice nurses were documented using a standard reporting form and reviewed by the Data Safety and Monitoring Board (DSMB).

### Statistical analysis

The target sample size was based on detecting a medium effect (0.5 SD; mean difference (MD) = 3.57 points on the ANU-ADRI) with 80% power and α = 0.05, assuming intraclass correlation coefficient (ICC) = 0.05 and an average cluster size of 10. The standard deviation was based on a previous ANU-ADRI intervention study conducted in a comparable primary care population in Australia.^25^ Allowing for 20% clinic attrition and 30% participant attrition, the required enrolment was 390 participants (195 per arm) across 26 clinics (13 per arm).

The sample-size calculation indicated that a minimum of 26 clinics (13 per group) was required. Operationally, additional clinics were recruited and randomised to account for anticipated clinic withdrawal and variability in participant recruitment and to ensure achievement of the required participant sample size.

Baseline characteristics were summarised using descriptive statistics. Given the cluster-randomised design, in which clinics were randomised before participant recruitment, baseline characteristics were compared between treatment groups to assess potential participant-level imbalances arising from differential recruitment after randomisation. Continuous variables were compared using independent-samples t tests or Mann–Whitney U tests, as appropriate, and categorical variables using chi-square or Fisher's exact tests.

Analyses were conducted in accordance with the published protocol.^8^ Outcomes were assessed at the participant level. The primary effectiveness analysis was according to an intention-to-treat (ITT) principle. Changes in ANU-ADRI scores over the 12-month follow-up period were assessed using a linear mixed-effects model fitted with maximum likelihood estimation. Model assumptions were assessed through visual inspection of residual histograms and normal Q–Q plots. Residuals were broadly symmetric, with modest departures from normality and some evidence of heavier tails. Given the sample size, linear mixed-effects models were considered robust to these departures. Where appropriate, change scores were transformed so that positive values consistently indicate improvement; accordingly, positive between-group MDs favour the intervention group. The prespecified analyses for the primary and secondary outcomes included clinic as a random intercept and treatment group, marital status, Indigenous status, socioeconomic status, Charlson Comorbidity Index (CCI), and hypertension as participant-level fixed-effect covariates measured at baseline. For outcomes in which a prespecified covariate represented or overlapped with a component used to derive the outcome score, that covariate was excluded from the corresponding model. Within-group changes were estimated using model-based estimated marginal means, and between-group differences were obtained from the same mixed-effects model. Type III F-tests with the Satterthwaite approximation for denominator degrees of freedom were used to derive 95% confidence intervals and p values. Missing 12-month outcome data were addressed using multiple imputation by chained equations under a missing-at-random assumption, conditional on the observed variables included in the imputation model. For the primary outcome, missing 12-month ANU-ADRI component variables were imputed before deriving the 12-month ANU-ADRI total, risk-factor, protective-factor, and change scores; change scores were not imputed directly. The imputation model included treatment allocation, clinic as a categorical predictor, all baseline ANU-ADRI component variables, together with selected baseline demographic and clinical variables (marital status, Indigenous status, socioeconomic status, Charlson Comorbidity Index, and hypertension). These variables were selected a priori based on their clinical relevance and plausible associations with the outcome and/or missing follow-up data. Twenty imputed datasets were generated, exceeding the proportion of participants with missing 12-month primary outcome data (18%). For secondary outcomes, imputation models included treatment allocation, clinic as a categorical predictor, the corresponding baseline and 12-month outcome variables, and relevant baseline demographic and clinical variables associated with the outcome or missingness. Each analysis model was fitted separately within every imputed dataset, and parameter estimates and their within- and between-imputation variances were combined using Rubin's rules^26^ to obtain pooled treatment-effect estimates, standard errors, 95% confidence intervals, and p values. Participants who died before the 12-month assessment were excluded from analyses of 12-month outcomes because these outcomes were undefined after death and were not imputed.

Complete-case analyses were conducted as sensitivity analyses to examine the robustness of results to the imputation approach. A post-hoc sensitivity analysis additionally adjusting for baseline ANU-ADRI score was performed to assess the robustness of the primary findings to the observed baseline imbalance.

Exploratory subgroup analyses examined consistency of intervention effects by age, sex, relationship status, socioeconomic status, baseline dementia risk, and number of risk factors targeted. Treatment-by-subgroup interaction tests were performed to formally assess evidence of effect modification.

Per-protocol analyses were also performed among participants who completed the intervention as intended. This was defined as attending the initial interview with the nurse (control group) and engaging in at least 4 of 5 intervention sessions in the 12-month intervention period and making entries in the mobile app for ≥13 weeks (intervention group). Participants meeting these adherence criteria comprised the per-protocol population. Missing outcome data were handled using the same multiple-imputation procedure as the primary ITT analysis, after which analyses were restricted to the per-protocol population within each imputed dataset. The per-protocol analysis was considered a supportive sensitivity analysis, with the ITT analysis remaining the primary analysis of intervention effectiveness.

No adjustment for multiple testing (e.g., Bonferroni correction) was prespecified or applied. As the trial was powered for the primary outcome, findings from secondary outcomes should therefore be considered exploratory and interpreted cautiously, particularly given the increased risk of Type I error arising from multiple comparisons.

Analyses were performed using Stata (version 19.0; StataCorp, College Station, TX, USA) and SPSS (version 30.0; IBM, Armonk, NY, USA). No interim analyses or stopping guidelines were specified a priori. A DSMB, comprising a general practitioner, nurse, dietitian, neuropsychologist, and exercise physiologist, reviewed all adverse events.

### Role of the funding source

The study was funded by the Australian Government National Health and Medical Research Council, Boosting Dementia Research Grants (NHMRC, GNT1171851). The study funder had no role in study design, data collection, analysis, interpretation, writing of the report, or the decision to submit for publication. JG (the corresponding author) had full access to all data and takes final responsibility for publication.

## Results

A total of 56 general practices were approached, of which 40 consented and were randomised (20 intervention and 20 control clinics). Following randomisation, nine clinics (seven intervention and two control) withdrew before participant recruitment commenced because of operational, governance, or staffing reasons. No participants were recruited and no intervention components were delivered at these clinics. Although block randomisation was used to maintain approximate balance between study groups, these withdrawals resulted in an unequal number of clinics contributing participants to each group. Consequently, 31 clinics (13 intervention and 18 control) contributed participants to the trial (Fig. 1). The participating clinics recruited a median of 13 participants (IQR 7–22; range 1–26) per clinic. Recruitment continued until the prespecified sample size was achieved, with participating clinics recruiting up to 30 participants where capacity allowed, resulting in similar participant numbers between the intervention and control groups despite the unequal number of contributing clinics. Between February 2022 and April 2023, 430 participants consented, of whom 403 completed the baseline ANU-ADRI and were enrolled (201 intervention; 202 control). At 12 months, retention was 78% (156 of 201) in the intervention group and 88% (178 of 202) in the control group. In the control group, 198 participants (98%) received the brief intervention, whereas in the intervention group 161 (80%) attended ≥4 sessions and 71 (35%) entered data into the HAPPI MIND app for ≥13 weeks. Reasons for dropout are shown in Fig. 1 and dropout rates were not different between groups (p = 0.0860). Seven participants experienced adverse events during 12-month follow-up, including two deaths (one per group) and five serious adverse events (four intervention; one control). Two serious adverse events in the intervention group (distress related to pre-existing disordered eating) were considered related to the intervention.

**Fig. 1:**
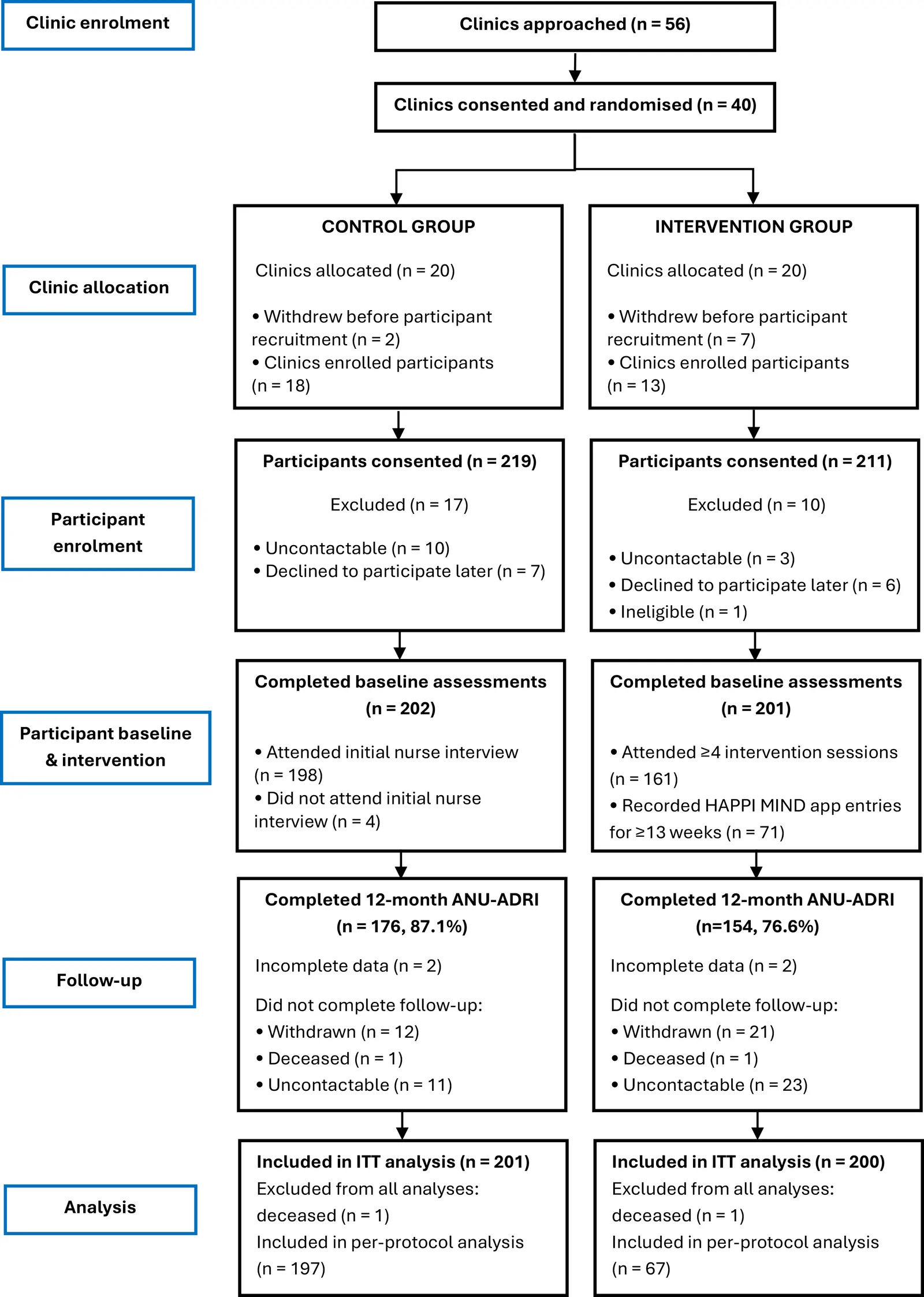
Holistic Approach in Primary Care for Preventing Memory Impairment and Dementia (HAPPI MIND) study flow diagram. **Note:** The numbers of individuals assessed for eligibility, approached, and declining participation were not routinely recorded by participating practices and are therefore unavailable.

Table 1 presents the participants baseline characteristics. Participants were predominantly female (63%) with a mean age of 56.4 years (SD 5.7). Most were Australian-born (81%) and employed (72%), with a median CCI score of 0.0 (IQR 0.0–1.0). Baseline cognitive and quality-of-life measures were: ANU-ADRI, mean 1.91 (SD 7.02); CAIDE, median 6.0 (IQR 5.0–7.0); MoCA, median 27.0 (IQR 25.0–29.0); and EQ-5D-5L index, median 0.92 (IQR 0.85–0.96). The median number of dementia risk factors was 5.0 (IQR 4.0–6.0).

**Table 1:** Characteristics of the study participants (n = 403).

Characteristics	Intervention, n = 201	Control, n = 202	Total, n = 403	p value
Age, mean (SD), years	56.6 (5.83)	56.3 (5.56)	56.4 (5.69)	0.62
Sex, (Female)	121 (60)	131 (65)	252 (63)	0.34
Born in Australia, (Yes)^a^	162 (81)	164 (81)	326 (81)	0.96
Mainly speaks English at home^b^	187 (96)	193 (97)	380 (96)	0.56
Marital status, (Living in a relationship)	140 (70)	131 (65)	271 (67)	0.31
Currently employed, (Yes)^a^	149 (75)	141 (70)	290 (72)	0.26
Body mass index (BMI), median [IQR]	32.2 [27.4–38.8]	30.8 [26.5–35.9]	31.5 [27.1–37.0]	**0.014**
Socioeconomic status, SEIFA-IRSAD (deciles)				
1–3 (low socioeconomic class)	17 (9)	40 (20)	57 (14)	**<0.0010**
4–6 (middle socioeconomic class)	88 (44)	37 (18)	125 (31)	
7–10 (high socioeconomic class)	96 (48)	125 (62)	221 (55)	
Aboriginal and/or Torres Strait Islander, (Yes)	12 (6)	14 (7)	26 (7)	0.70
Geographic location−ARIA+, Major city, (Yes)	152 (76)	157 (78)	309 (77)	0.29
Number of prescribed medicines, median (IQR)^c^	2.5 [1.0–4.0]	2.0 [1.0–4.0]	2.0 [1.0–4.0]	0.71
CCI score, median (IQR)^d^	0.0 [0.0–1.0]	0.0 [0.0–1.0]	0.0 [0.0–1.0]	0.85
ANU-ADRI Total score, mean (SD)	2.56 (7.14)	1.25 (6.86)	1.91 (7.02)	0.061
CAIDE score, median (IQR)^e^	6.0 [5.0–8.0]	6.0 [5.0–7.0]	6.0 [5.0–7.0]	0.17
MoCA score, median (IQR)^f^	27.0 [25.0–29.0]	27.0 [26.0–29.0]	27.0 [25.0–29.0]	0.27
EQ-5D-5L index score, median (IQR)	0.92 [0.85–0.96]	0.92 [0.82–0.96]	0.92 [0.85–0.96]	0.15
EQ-5D-5L VAS, median (IQR)	71.0 [50.0–80.5]	70.0 [50.0–80.0]	70.0 [50.0–80.0]	0.19
Number of dementia risk factors, median (IQR)	5.0 [4.0–6.0]	5.0 [3.0–6.0]	5.0 (4.0–6.0)	0.15

***Note***: Data are presented as n (%), mean (SD) or median (IQR).

Bold text indicates statistically significant results (p < 0.05).

**ANU-ADRI**, Australian National University Alzheimer's Disease Risk Index; **ARIA**, Accessibility/Remoteness Index of Australia; **CAIDE**, Cardiovascular Risk Factors, Ageing, and Incidence of Dementia; **CCI**, Charlson comorbidity index; **EQ-5D-5L VAS**, EuroQol 5-Dimension 5-Level Visual Analogue Scale; **IQR**, Interquartile range (25th–75th percentile); **MoCA**, Montreal Cognitive Assessment; **SEIFA-IRSAD**, Socioeconomic Indexes for Areas–Index of Relative Socioeconomic Advantage and Disadvantage; **SD**, Standard deviation.

a Data were missing for 2 participants.

b Data were missing for 9 participants.

c Data were missing for 6 participants.

d Data were missing for 5 participants.

e Data were missing for 35 participants.

f Data were missing for 15 participants.

Baseline ANU-ADRI scores were numerically higher in the intervention group than in the control group; however, this difference was not statistically significant (p = 0.061). A sensitivity analysis additionally adjusting for baseline ANU-ADRI score was performed to assess the robustness of the primary findings (Table 2).

**Table 2:** Change in the ANU-ADRI scores from baseline to 12-months–adjusted and unadjusted analyses.

Variable	Within-group difference		Between-group difference^a^	% Showing improvement
	Intervention, n = 200	Control, n = 201		Intervention/Control
Adjusted analyses (MD, 95% CI, p value)				
ANU-ADRI total score (ITTA−*Primary outcome;* n = 401)	0.791 (−0.895 to 2.478)	−0.807 (−2.339 to 0.725)	**1.598 (0.423–2.773), 0.0080**	56/44
ANU-ADRI risk factor sub-score (n = 401)	**−1.392 (−2.677 to −0.108)**	**−1.436 (−2.543 to −0.328)**	0.043 (−0.826 to 0.912), 0.92	25/28
ANU-ADRI protective factor sub-score (n = 401)	**2.196 (0.559–3.833)**	0.638 (−0.616 to 1.892)	**1.558 (0.439–2.678), 0.0070**	61/47
ANU-ADRI total score (post-hoc sensitivity analysis—additionally adjusted for baseline ANU-ADRI score; n = 401)	0.200 (−1.422 to 1.821)	−0.979 (−2.454 to 0.496)	**1.179 (0.039–2.320), 0.043**	56/44
ANU-ADRI total score (Per-protocol analysis; n = 264)	1.261 (−0.604 to 3.126)	−1.210 (−2.792 to 0.371)	**2.471 (0.806–4.137), 0.0040**	61/44
ANU-ADRI total score (Complete case analysis; n = 330)	0.741 (−0.665 to 2.146)	−0.507 (−1.871 to 0.856)	**1.248 (0.032–2.464), 0.045**	56/45
Unadjusted analyses (MD, 95% CI, p value)				
ANU-ADRI total score (ITTA−*Primary outcome;* n = 401)	**1.675 (0.650–2.699)**	0.106 (−0.796 to 1.008)	**1.569 (0.442–2.696), 0.0060**	56/44
ANU-ADRI risk factor sub-score (n = 401)	**−0.813 (−1.585 to −0.041)**	**−0.860 (−1.475 to −0.245)**	0.047 (−0.835 to 0.929), 0.92	25/28
ANU-ADRI protective factor sub-score (n = 401)	**2.488 (1.323–3.653)**	**0.966 (0.217–1.716)**	**1.522 (0.456–2.588), 0.0050**	61/47
ANU-ADRI total score (Per-protocol analysis; n = 264)	**2.485 (1.142–3.828)**	0.071 (−0.798 to 0.940)	**2.414 (0.821–4.007), 0.0030**	61/44
ANU-ADRI total score (Complete case analysis; n = 330)	**1.682 (0.880–2.483)**	0.483 (−0.267 to 1.233)	**1.20 (0.101–2.296), 0.032**	56/45

**Note:** Deceased participants (n = 2) were excluded from the analyses. Bold text indicates statistically significant results (p < 0.05).

**ANU-ADRI**, Australian National University Alzheimer's Disease Risk Index; **ITTA**, Intention-to-treat analysis; **MD**, Mean difference.

a Change scores were transformed, where appropriate, so that positive values consistently indicated improvement (i.e., reduced dementia risk). For the ANU-ADRI total score, risk-factor sub-score, and protective-factor sub-score, positive values indicate reduced dementia risk or a more favourable dementia risk profile.

The prevalence of dementia risk factors was comparable between groups (Supplementary Table S2). Uncontrolled hyperlipidaemia (93%), low cognitive activity (73%), and obesity (58%) were the most prevalent risk factors while physical inactivity and uncontrolled diabetes were more prevalent in the intervention group and, the remaining risk factors were broadly similar between groups.

Two participants (one in each study group) died before the 12-month assessment and were therefore excluded from the 12-month outcome analyses. The ITT analysis included 401 participants, whereas the per-protocol analysis included 264 participants (67 intervention and 197 control) who met the per-protocol criteria. The substantially smaller intervention per-protocol population reflected the requirement to meet both the nurse-session and mobile-app engagement criteria. A positive change in ANU-ADRI score reflects a reduction in estimated dementia risk (i.e., a more favourable risk profile).

In both adjusted and unadjusted analyses, the intervention group demonstrated a statistically significant greater improvement in the primary outcome (ANU-ADRI total score) compared with the control group (Table 2). Overall, 56% of participants in the intervention group and 44% in the control group showed improvement in the primary outcome. In the adjusted ITT analysis, the between-group mean difference (MD) was 1 1.60 (95% CI 0.42–2.77; p = 0.0080), while the unadjusted MD was 1.57 (95% CI 0.44–2.70; p = 0.0060), favouring the intervention. Findings were consistent in per-protocol analyses (adjusted MD 2.47, 95% CI 0.81–4.14; p = 0.0040; unadjusted MD 2.41, 95% CI 0.82–4.01; p = 0.0030) and complete-case analysis (adjusted MD 1.25, 95% CI 0.03–2.46; p = 0.045; unadjusted MD 1.20, 95% CI 0.10–2.30; p = 0.032). In the sensitivity analysis additionally adjusting for baseline ANU-ADRI score, the between-group difference remained significant (adjusted MD 1.18, 95% CI 0.04–2.32; p = 0.043), consistent with the primary protocol-specified analysis (Table 2).

Changes in the ANU-ADRI risk factor sub-score were minimal and there were no statistically significant differences between groups in either adjusted (MD 0.04, 95% CI –0.83 to 0.91; p = 0.92) or unadjusted analysis (MD 0.05, 95% CI –0.84 to 0.93; p = 0.92). In contrast, the ANU-ADRI protective factor sub-score improved significantly more in the intervention group than in the control group in both the adjusted (MD 1.56, 95% CI 0.44–2.68; p = 0.0070) and unadjusted analyses (MD 1.52, 95% CI 0.46–2.59; p = 0.0050).

The baseline and 12-month distributions of the individual ANU-ADRI components are presented in Supplementary Table S3. Adjusted analyses of individual ANU-ADRI component change scores showed significant improvements in the intervention group than in the control group for physical activity (adjusted MD 0.49, 95% CI 0.13–0.84; p = 0.0080), fish intake (adjusted MD 0.54, 95% CI 0.13–0.96; p = 0.010), and BMI status (adjusted MD 0.36, 95% CI 0.02–0.71; p = 0.041) (Table 3).

**Table 3:** Changes in individual ANU-ADRI component scores from baseline to 12 months.

ANU-ADRI component	Within-group difference		Between-group difference	% Showing improvement^a^
	Intervention (n = 200)	Control (n = 201)	Adjusted MD (95% CI), p value	Intervention/Control
Physical activity	0.438 (−0.137 to 1.013)	−0.047 (−0.441 to 0.347)	**0.485 (0.127–0.843), 0.0080**	38/26
Cognitive activity	**1.175 (0.377–1.973)**	0.703 (−0.063 to 1.470)	0.472 (−0.219 to 1.162), 0.18	26/21
Fish intake	**0.753 (0.250–1.257)**	0.210 (−0.235 to 0.655)	**0.543 (0.128–0.958), 0.010**	34/22
Alcohol intake	−0.209 (−1.396 to 0.978)	−0.280 (−1.089 to 0.529)	0.072 (−0.520 to 0.663), 0.81	8/11
Smoking	0.006 (−0.124 to 0.136)	−0.020 (−0.146 to 0.105)	0.026 (−0.101 to 0.154), 0.68	3/1
BMI status	0.493 (−0.072 to 1.058)	0.131 (−0.334 to 0.596)	**0.362 (0.015–0.709), 0.041**	15/11
Depression	−0.049 (−0.371 to 0.272)	−0.002 (−0.276 to 0.273)	−0.048 (−0.254 to 0.158), 0.65	13/19
Social engagement	−0.361 (−1.083 to 0.361)	−0.205 (−0.759 to 0.348)	−0.156 (−0.611 to 0.299), 0.50	17/20
Age and sex score	**−0.241 (−0.468 to −0.013)**	**−0.289 (−0.512 to −0.066)**	0.049 (−0.149 to 0.246), 0.63	–
High cholesterol	0.059 (−0.143 to 0.261)	−0.009 (−0.204 to 0.185)	0.069 (−0.100 to 0.237), 0.42	–
Diabetes	**−0.344 (−0.543 to −0.145)**	**−0.353 (−0.560 to −0.145)**	0.009 (−0.152 to 0.169), 0.92	–
Traumatic brain injury	**−0.767 (−1.510 to −0.024)**	−0.503 (−1.021 to 0.015)	−0.264 (−0.614 to 0.086), 0.14	–
Occupational pesticide exposure	−0.157 (−0.397 to 0.082)	−0.126 (−0.311 to 0.058)	−0.031 (−0.148 to 0.086), 0.60	–

***Note:*** Components representing fixed characteristics or lifetime diagnoses or exposures (e.g., age and sex, education, diabetes, high cholesterol, traumatic brain injury, and occupational pesticide exposure) have limited or no capacity for improvement over the 12-month follow-up period; therefore, percentages showing improvement are not presented for these components. Detailed descriptions of the ANU-ADRI variables, component categories, and scoring algorithm are provided in Supplementary File 2.

Bold text indicates statistically significant results (p < 0.05).

**ANU-ADRI**, Australian National University Alzheimer's Disease Risk Index; **BMI**, Body mass index; **CI**, Confidence interval; **MD**, Mean difference.

a Percentage of participants whose individual ANU-ADRI component score improved from baseline to 12 months. Positive MDs favour the intervention group and indicate improvement relative to the control group. Adjusted between-group differences were estimated using the same linear mixed-effects modelling approach as for the primary outcome, accounting for clustering by clinic and adjusting for prespecified baseline covariates.

Table 4 summarises changes in secondary outcomes from baseline to 12 months. No statistically significant between-group differences were observed for any of the secondary outcomes. No participants were reported to have received a diagnosis of dementia during the 12-month follow-up period, and changes in MoCA scores were minimal with no significant between-group differences observed. Longer-term cognitive and clinical outcomes will be examined in the ongoing 36-month follow-up.

**Table 4:** Change in the secondary outcomes from baseline to 12-months (MD, 95% CI, p value).

Variable	Within-group difference		Between-group difference	% Showing improvement^a^
	Intervention, n = 200	Control, n = 201		Intervention/Control
CAIDE	−0.197 (−0.558 to 0.164)	**−0.359 (−0.681 to −0.038)**	0.162 (−0.090 to 0.415), 0.21	29/28
MoCA	0.325 (−0.271 to 0.922)	0.214 (−0.368 to 0.796)	0.111 (−0.395 to 0.617), 0.67	51/49
AUSDRISK	0.345 (−0.500 to 1.189)	0.515 (−0.285 to 1.315)	−0.170 (−0.975 to 0.635), 0.68	46/45
Absolute CVD risk	−0.612 (−1.262 to 0.039)	**−0.746 (−1.336 to −0.156)**	0.134 (−0.422 to 0.691), 0.63	32/30
TABS	**−1.214 (−2.269 to −0.159)**	**−1.403 (−2.443 to −0.363)**	0.189 (−0.738 to 1.116), 0.69	36/38
MIND diet	**−1.433 (−2.139 to −0.726)**	**−1.178 (−1.866 to −0.490)**	−0.255 (−0.893 to 0.382), 0.43	38/37
PAM	1.593 (−2.312 to 5.499)	0.459 (−3.364 to 4.283)	1.134 (−2.236 to 4.505), 0.51	60/54
AUDIT	**0.730 (0.138–1.322)**	0.278 (−0.305 to 0.861)	0.452 (−0.056 to 0.960), 0.081	46/47
SCQ	0.445 (−0.286 to 1.176)	0.138 (−0.580 to 0.857)	0.307 (−0.348 to 0.961), 0.36	54/46
EQ-5D-5L	0.006 (−0.044 to 0.057)	0.016 (−0.037 to 0.069)	−0.009 (−0.054 to 0.036), 0.69	39/40
EQ-5D-5L VAS	−0.347 (−4.085 to 3.391)	0.065 (−3.613 to 3.744)	−0.413 (−3.653 to 2.828), 0.80	49/49
PROMIS	**1.615 (0.367–2.864)**	0.825 (−0.407 to 2.056)	0.791 (−0.287 to 1.869), 0.15	53/44
STOP-BANG	0.206 (−0.063 to 0.475)	0.005 (−0.261 to 0.272)	0.201 (−0.013 to 0.415), 0.065	40/27
ISI	0.235 (−0.818 to 1.288)	0.201 (−0.838 to 1.240)	0.035 (−0.862 to 0.931), 0.94	45/48

**Note:** Deceased participants (n = 2) were excluded from the analyses. Adjusted estimates were obtained from linear mixed-effects models including clinic as a random intercept and treatment group, marital status, Indigenous status, socioeconomic status, Charlson Comorbidity Index, and hypertension as fixed-effect covariates. Analyses were based on the multiply imputed datasets, with parameter estimates pooled using Rubin's rules. No adjustment for multiple testing was applied. Bold text indicates statistically significant results (p < 0.05).

**AUDIT**, Alcohol Use Disorders Identification Test; **AUSDRISK**, Australian Type 2 Diabetes Risk Assessment Tool; **CAIDE**, Cardiovascular Risk Factors, Aging, and Incidence of Dementia; **EQ-5D-5L VAS**, EuroQol 5-Dimension 5-Level Visual Analogue Scale; **ISI**, Insomnia Severity Index; **MIND**, Mediterranean-DASH Intervention for Neurodegenerative Delay; **MoCA**, Montreal Cognitive Assessment; **PAM**, Patient Activation Measure; **PROMIS**, Patient-Reported Outcomes Measurement Information System; **SCQ**, Self-Administered Comorbidity Questionnaire; **STOP-BANG**, STOP-BANG Sleep Apnoea Questionnaire; **TABS**, Tool for Adherence Behaviour Screening.

a Improvement was defined as a change from baseline to 12 months in the favourable direction for each outcome.

For within-group changes, the intervention group showed significant improvements in satisfaction with participation in social and leisure activities (PROMIS; MD 1.62, 95% CI 0.37–2.86) and harmful alcohol use (AUDIT; MD 0.73, 95% CI 0.14–1.32) in the intervention group. However, MIND diet (MD −1.43, 95% CI −2.14 to −0.73) and medication adherence behaviour (TABS; MD −1.21, 95% CI −2.27 to −0.16) scores declined over 12 months.

In contrast, the control group demonstrated significant within-group changes for absolute CVD risk (MD −0.75, 95% CI −1.34 to −0.16), TABS (MD in addition to reductions in medication adherence behaviour (TABS; MD −1.40, 95% CI −2.44 to −0.36), and MIND diet (MD −1.18, 95% CI −1.87 to −0.49), indicating worsening cardiovascular risk, treatment adherence, and dietary behaviours over time.

Across all secondary outcomes, the proportion of participants showing improvement ranged from 29% to 60% in the intervention group and from 27% to 54% in the control group.

Subgroup analyses examining within-group and between-group differences across participant characteristics are presented in Table 5. Formal tests of interaction showed evidence of treatment-effect heterogeneity by socioeconomic status (p-interaction = 0.0340) and baseline dementia risk (p-interaction = 0.0040). For socioeconomic status, the between-group MD was 3.824 (95% CI −0.774 to 8.421) in the low socioeconomic group, 1.998 (−0.210 to 4.205) in the middle socioeconomic group, and 0.288 (−1.158 to 1.734) in the high socioeconomic group. For baseline dementia risk, the between-group MD was 3.769 (1.355–6.182) among participants with high baseline dementia risk (ANU-ADRI score ≥5) and 0.068 (−1.167 to 1.304) among those with lower baseline dementia risk (ANU-ADRI score <5). There was no evidence of interaction by age, sex, marital status, Australian-born status, or number of dementia risk factors at baseline (all p-interaction>0.05).

**Table 5:** Change in the ANU-ADRI total score from baseline to 12-months (MD, 95% CI, p value)—adjusted subgroup analyses.

Subgroup (Total n; Intervention/Control)	Within-group difference		Between-group difference^a^	% Showing improvement	p value for interaction
	Intervention	Control		Intervention/Control	
45–59 years (n = 262; 129/133)	0.906 (−1.293 to 3.104)	−1.325 (−3.346 to 0.695)	**2.231 (0.630–3.832), 0.0060**	58/43	0.21
≥60 years (n = 139; 71/68)	1.222 (−0.858 to 3.302)	0.592 (−1.462 to 2.647)	0.629 (−1.038 to 2.297), 0.46	53/46	
Female (n = 250; 120/130)	0.081 (−1.955 to 2.118)	−1.674 (−3.570 to 0.223)	**1.755 (0.098–3.413), 0.038**	57/45	0.89
Male (n = 151; 80/71)	1.825 (−0.497 to 4.146)	−0.002 (−2.258 to 2.253)	**1.827 (0.088–3.566), 0.039**	55/41	
Australian-Born (n = 324; 161/163)	1.114 (−0.925 to 3.152)	−0.546 (−2.306 to 1.214)	**1.659 (0.347–2.971), 0.013**	59/45	0.69
Not Australian-born (n = 75; 37/38)	−0.812 (−3.631 to 2.007)	−2.105 (−4.968 to 0.758)	1.293 (−1.231 to 3.817)	41/37	
Married (Living in a relationship = yes) (n = 271; 140/131)	0.536 (−1.277 to 2.350)	−0.667 (−2.380 to 1.047)	1.203 (−0.118 to 2.524), 0.074	55/45	0.33
Single (n = 130; 60/70)	1.375 (−1.115 to 3.864)	−1.054 (−3.437 to 1.329)	**2.429 (0.188–4.670), 0.034**	58/42	
Low socioeconomic class (SEIFA-IRSAD: 1−3) (n = 56; 17/39)	0.529 (−4.414 to 5.473)	−3.294 (−7.346 to 0.757)	3.824 (−0.774 to 8.421), 0.10	53/34	**0.034**
Middle socioeconomic class (SEIFA-IRSAD: 4−6) (n = 125; 88/37)	1.410 (−0.837 to 3.657)	−0.588 (−3.071 to 1.896)	1.998 (−0.210 to 4.205), 0.076	58/36	
High socioeconomic class (SEIFA-IRSAD: 7−10) (n = 220; 95/125)	−0.215 (−2.119 to 1.689)	−0.503 (−2.317 to 1.310)	0.288 (−1.158 to 1.734). 0.70	55/49	
Dementia risk at baseline (ANU-ADRI score ≥5) (n = 141; 78/63)	**3.621 (1.052–6.190)**	−0.147 (−2.583 to 2.288)	**3.769 (1.355–6.182), 0.0020**	76/52	**0.0040**
Dementia risk at baseline (ANU-ADRI score <5) (n = 260; 122/138)	−1.358 (−3.161 to 0.444)	−1.427 (−3.121 to 0.267)	0.068 (−1.167 to 1.304), 0.91	43/40	
Dementia risk factors at baseline ≥4 (n = 301; 157/144)	1.561 (−0.424 to 3.547)	−0.141 (−2.011 to 1.729)	**1.702 (0.099–3.305), 0.037**	60/51	0.38
Dementia risk factors at baseline <4 (n = 100; 43/57)	−1.851 (−4.335 to 0.633)	−2.240 (−4.878 to 0.399)	0.389 (−1.565 to 2.343), 0.70	43/27	
**Intervention group only**	**Yes (n = 145)**	**No (n = 55)**	**Between-group difference**		
Risk factors targeted ≥4	1.715 (−0.123 to 3.553)	0.340 (−3.028 to 3.708)	1.376 (−1.169 to 3.920), 0.28	57/52	–

**Note:** Deceased participants (n = 2) were excluded from the analyses. Values in parentheses represent the total number of participants contributing to each subgroup analysis, followed by the numbers in the intervention and control groups, respectively. Bold text indicates statistically significant results (p < 0.05).

**ANU-ADRI**, Australian National University Alzheimer's Disease Risk Index; **MD**, Mean difference; **SEIFA-IRSAD**, Socioeconomic Indexes for Areas–Index of Relative Socioeconomic Advantage and Disadvantage.

a Change scores were transformed, where appropriate, so that positive values consistently indicate improvement (i.e., a more favourable dementia risk profile). “No” refers to intervention participants in whom fewer than four modifiable dementia risk factors were actively targeted during the intervention period.

Supplementary Table S4 summarises changes in dementia risk-factor clinical and biomarker measures from baseline to 12 months. No statistically significant between-group differences were observed for any of these measures. Within-group analyses showed significant improvements in low-density lipoprotein (LDL) cholesterol in both groups, including among participants with baseline LDL >2.0 mmol/L. Total cholesterol also improved significantly in both groups among participants with baseline levels >6.5 mmol/L. Among participants with elevated blood pressure at baseline, significant improvements in diastolic blood pressure were observed in both groups, whereas systolic blood pressure improved significantly in the control group only. In the intervention group, 14 modifiable dementia risk factors were targeted by nurses, and most frequently addressed risk factors were physical inactivity (80%), overweight or obesity (71%), and low cognitive activity (69%). When directly comparing participants in whom each risk factor was targeted with those in whom the same risk factor was not targeted, a significantly greater improvement in ANU-ADRI total score was observed for social isolation (MD 3.64, 95% CI 0.78–6.49; p = 0.013; Supplementary Table S5).

## Discussion

This pragmatic clinical trial, found a modest but statistically significant improvement in estimated dementia-risk profiles, as measured by the ANU-ADRI, at 12 months compared with the active control group. The intervention appeared more effective in improving protective lifestyle-related behaviours than in reducing dementia risk factors. After adjusting for confounding factors not captured by the ANU-ADRI, the intervention arm demonstrated a greater mean reduction of 1.6 units in the ANU-ADRI score compared to the usual care arm, where higher scores are associated with greater estimated dementia risk in observational studies.^27^

The improvement in total ANU-ADRI score appeared to be driven primarily by modest cumulative changes across multiple modifiable behavioural components rather than large changes in individual risk factors alone. Component-level analyses showed improvements in physical activity, fish intake, and BMI status in the intervention group compared with the control group. These findings are consistent with the multidomain design of the intervention, which targeted several modifiable dementia risk factors simultaneously through personalised behavioural counselling and goal-setting approaches.

The magnitude of change in ANU-ADRI observed in this study is broadly consistent with, but somewhat smaller than, that reported in previous multidomain intervention trials.^6^, ^24^, ^25^ These findings suggest that a nurse-led multidomain dementia risk-reduction intervention can achieve modest improvements in estimated dementia risk profiles within Australian primary care. The HAPPI MIND intervention allowed participants to personalise the specific risk or protective factors they wished to target, with support from a nurse within their usual care setting, enhancing its practicality and relevance to real-world implementation. Exploratory subgroup analyses suggested potential heterogeneity in intervention effects according to baseline dementia risk and socioeconomic status, with formal interaction tests reaching statistical significance for both. However, these findings should be interpreted cautiously because the trial was not powered to detect subgroup interactions and multiple exploratory comparisons were undertaken. They should therefore be considered hypothesis-generating and require confirmation in adequately powered studies. Additional analyses from the HAPPI MIND program, including 36-month follow-up outcomes, qualitative evaluations of participant and nurse experiences, intervention acceptability, and satisfaction with the model of care, will be reported separately.

Many of the modifiable risk factors targeted in HAPPI MIND overlap with those routinely addressed within chronic disease prevention and management programs in primary care, including cardiovascular disease, diabetes, obesity, smoking, and physical inactivity. Integration of dementia-risk reduction strategies within existing chronic disease management and preventive health frameworks may provide a more efficient and sustainable approach for implementation in resource-constrained health-care settings.

Potential mechanisms underlying the intervention's effect include increased awareness of dementia risk, personalised feedback via the ANU-ADRI report, and structured behavioural support using motivational interviewing and goal-setting, complemented by mHealth-enabled self-monitoring/management. However, these processes may also contribute to increased behavioural awareness and reporting of healthy behaviours, particularly for self-reported outcomes using tools such as the ANU-ADRI.

Despite improvements in estimated dementia-risk profiles, there were limited between-group differences in objective clinical outcomes over 12 months. Most observed improvements were seen in self-reported behavioural domains, including protective lifestyle-related factors, which may have been influenced by reporting bias or increased behavioural awareness associated with repeated assessments and behavioural coaching. Both the intervention and control groups demonstrated modest declines in MIND diet and medication adherence (TABS) scores over the 12-month follow-up, with no significant between-group differences, suggesting that these behaviours were not improved by the nurse-led intervention. Dietary modification and medication adherence may require more targeted, discipline-specific support, and future multidomain dementia prevention interventions could incorporate input from dietitians and pharmacists to specifically address these areas. The improvement in blood pressure observed in the control group may reflect ongoing pharmacological management and routine clinical care provided by general practitioners during the study period. In addition, all participants, including controls, received a minimal intervention comprising an individualised ANU-ADRI dementia-risk report and brief risk-reduction advice, which may have increased awareness of vascular risk factors and prompted further discussion and management with their general practitioners. These findings highlight the complexity of addressing multiple dementia risk factors through a single intervention model and suggest that incorporating multidisciplinary expertise may strengthen future interventions.

This trial has several strengths. It is, to our knowledge, the first cluster randomised controlled trial to evaluate a multidomain, nurse-led, mHealth-supported dementia-risk reduction program specifically targeting middle-aged adults in Australian primary care. The pragmatic design enhances generalisability, and the inclusion of validated behavioural and clinical measures allows a comprehensive assessment of dementia-risk change. Outcome assessors and data analysts were blinded, reducing measurement bias, and multiple imputation was used to address missing data. The magnitude and consistency of benefit across the ITT, per-protocol, and complete-case analyses increase confidence in our findings.

Limitations include the open-label nature of the intervention, which could introduce performance or reporting bias despite partial disclosure and blinded outcome assessments. Participant recruitment occurred after clinic randomisation; hence, the study was also susceptible to recruitment bias if knowledge of clinic allocation influenced participant enrolment. However, several measures were implemented to minimise this risk. The primary outcome is largely derived from self-reported behaviours and may therefore be susceptible to social desirability, reporting bias, and intervention-induced awareness effects, particularly in the context of an unblinded behavioural intervention. The absence of consistent changes in objective clinical outcomes suggests that some of the observed effects may reflect increased awareness or changes in reporting rather than true behavioural modification.

The 12-month follow-up captured early effects, and sustainability of behavioural change remains to be established. Future 36-month data analyses will determine whether these early gains are sustained and whether benefits extend to improved cognitive function and/or other clinical outcomes. As observed in previous multidomain and digital health interventions, sustained adherence to behavioural and digital components may decline over time, highlighting the importance of ongoing follow-up and implementation evaluation. Although reductions in ANU-ADRI scores suggest a more favourable dementia risk profile, this measure is based on risk estimation rather than clinical outcomes, and changes in score may not directly translate into a reduction in dementia incidence. In addition, the ANU-ADRI applies differential weighting to individual risk and protective factors; consequently, improvements in more heavily weighted components may contribute disproportionately to changes in the overall score, and this should be considered when interpreting the magnitude of the observed intervention effect. Nevertheless, prevention modelling suggests that even modest reductions in midlife dementia risk factors may yield meaningful population-level benefit.^1^

The relatively modest baseline estimated dementia risk of the study population may also have limited the scope for further improvement in ANU-ADRI scores. This is consistent with our previously published latent class analysis of the HAPPI MIND cohort, which demonstrated heterogeneity in baseline dementia risk profiles, including a substantial subgroup characterised by lower overall risk and healthier behaviours.^28^ Future trials could consider recruiting participants with higher baseline estimated dementia risk or a greater burden of modifiable dementia risk factors, while balancing recruitment feasibility and the generalisability of findings to broader primary care populations.

As with all multiple-imputation approaches, our analyses assumed that outcome data were missing at random conditional on the observed variables included in the imputation model. Although this assumption cannot be verified empirically and missing-not-at-random mechanisms cannot be excluded, the consistency of the findings across the multiply imputed, complete-case, and per-protocol analyses supports the robustness of the primary conclusions. Finally, the trial was conducted in Australian general practices; replication in other health-care contexts and economic evaluation will be important to assess external validity and cost-effectiveness, respectively.

Although the intervention was designed to align with routine primary care delivery, it still required structured nurse training, repeated motivational interviewing sessions, and ongoing digital support, which may present implementation and resource challenges outside a trial setting. Engagement with the mHealth application was also variable, suggesting that sustained digital participation may be challenging to achieve in routine practice. The intervention was evaluated as an integrated nurse-led multidomain programme; thus it was not possible to determine the independent contribution of the mHealth component to the observed intervention effects. Consequently, whether sustained app engagement was a key driver of effectiveness remains uncertain. Future research should optimise digital engagement, examine component-specific engagement and effects, and undertake implementation and economic evaluations to determine the feasibility, scalability, cost-effectiveness, and long-term sustainability of this model of care.

In conclusion, a multidomain intervention led by practice nurses and supported by mHealth technology achieved modest short-term improvements in estimated dementia risk profiles among middle-aged adults in Australian primary care. The intervention combined personalised dementia-risk feedback, targeted behavioural counselling, and digital self-monitoring: however, the independent contribution of the mHealth component could not be determined because the intervention was evaluated as an integrated package. This hybrid delivery model may inform future primary care interventions, although workforce capacity, training requirements, digital engagement, infrastructure, cost-effectiveness, and long-term sustainability require further evaluation before broader implementation. The ongoing 36-month follow-up will clarify the durability of these changes, their impact on cognitive and vascular outcomes, and the implementation strategies needed to extend reach to socioeconomically diverse and rural populations.

## Contributors

JG conceived the research idea and developed it with input from other chief investigators (PM, ALB, BB, SW, AM, VV, JSB, KM, SOR) and associate investigators (AJC, AH, DT, EM, KJA, MV, RJ, RAE, CYL). AJC developed the study protocol. KG was responsible for trial operations and data collection. GMG, RJ, MV were involved in the mobile application development. MRS accessed and curated the underlying data and performed the statistical analyses with methodological input from VV and JG. MRS draughted the first version of the manuscript. All authors contributed to data interpretation, critically reviewed and revised the manuscript, and approved the final version for submission. VV and TF directly accessed and verified the underlying data and vouch for the accuracy and integrity of the analyses. All authors had full access to all the data in the study and had final responsibility for the decision to submit for publication.

## Data sharing statement

Deidentified individual participant data (and a data dictionary) from this trial may be shared for ethically approved research purposes only, following submission of a study proposal and completion of a data access agreement. Requests can be made through the corresponding author at johnson.george@monash.edu.

## Declaration of interests

AJC is supported by a National Health and Medical Research Council (NHMRC) emerging leadership 1 grant (GNT2009633) and has received unrelated grant funding or consulting funding from the Medical Research Future Fund, NHMRC, Dementia Australia Research Foundation and the Pharmaceutical Society of Australia. All funds were paid to the employing institution. AJC also declares she is a national board director for the Pharmaceutical Society of Australia. ALB was supported by an NHMRC Fellowship (APP1135901, 2018–2022). VV is funded by the Rural Health Multidisciplinary Training Program. JSB has received grant funding or consulting funds from the National Health and Medical Research Council, Medical Research Future Fund, Victorian Government Department of Health and Human Services, Dementia Australia Research Foundation, Yulgilbar Foundation, Aged Care Quality and Safety Commission, Dementia Centre for Research Collaboration, Pharmaceutical Society of Australia, Advanced Pharmacy Australia, GlaxoSmithKline Supported Studies Programme, Amgen and several aged care provider organisations unrelated to this work. All grants and consulting funds were paid to the employing institution. DT has received grant funding from GlaxoSmithKline, unrelated to this work and paid to his employer. KJA is funded by ARC Laureate Fellowship (FL19010001). JG has received honoraria from GlaxoSmithKline, AstraZeneca and Pfizer for consultancy and educational/research grants from Boehringer-Ingelheim, GlaxoSmithKline and Pfizer for unrelated projects, all of which have been paid to his employer. All other authors declare no competing interests.
