# Supplementary material for: A multidomain, nurse-led, mobile health-supported intervention for dementia risk reduction in primary care (HAPPI MIND): a pragmatic, single-blind, two-arm, cluster randomised controlled trial
Source: Lancet Reg Health West Pac. 2026 Oct 20;75:102000. doi: 10.1016/j.lanwpc.2026.102000 (PMC13717166; doi:10.1016/j.lanwpc.2026.102000)
Supplement: Supplementary file 1 [file mmc3.pdf]

ANU-ADRI VARIABLES

A. *Protective (lowers score)*

1. Physical activity
   - 0 Low
   - -2 Medium
   - -3 High

2. Cog activity
   - 0 Low
   - -6 Medium
   - -7High

3. Fish intake
   - 0 Low
   - -3 Low-moderate
   - -4 Moderate
   - -5 High

B. *Mixed impact*

4. Alcohol
   - 3 Excessive
   - 0 None
   - -3 High

C. *Risk (raises score)*

5. Cholesterol
   - 0 No or age 60+
   - 3 Yes

6. Diabetes
   - 0 No
   - 3 Yes

7. BMI
   - 0 Normal or age 60+
   - 2 Overweight
   - 5 Obese

8. Smoking
   - 0 Never
   - 1 Ex-smoker
   - 4 Current smoker

9. Depression
   - 0 No
   - 2 Yes

10. Social
    - 0 High
    - 1 Medium-high
    - 4 Low-medium
    - 6 Low

11. Education
    - 0 High
    - 3 Medium
    - 6 Low

12. Traumatic Brain Injury
    - 0 No
    - 4 Yes

13. Occupational toxin exposure
    - 0 No
    - 2 Yes

14/15. Age and sex
- 0 Under 65 years
- 165+ male
- 5 65+ female
