# Supplementary material for: A multidomain, nurse-led, mobile health-supported intervention for dementia risk reduction in primary care (HAPPI MIND): a pragmatic, single-blind, two-arm, cluster randomised controlled trial
Source: Lancet Reg Health West Pac. 2026 Oct 20;75:102000. doi: 10.1016/j.lanwpc.2026.102000 (PMC13717166; doi:10.1016/j.lanwpc.2026.102000)
Supplement: Supplementary file 2 [file mmc1.docx]

**Supplementary Figure 1. HAPPI MIND model of care.** Reproduced from Cross AJ, Geethadevi GM, Magin P, Baker AL, Bonevski B, Godbee K, et al. A novel, multidomain, primary care nurse-led and mHealth-assisted intervention for dementia risk reduction in middle-aged adults (HAPPI MIND): study protocol for a cluster randomised controlled trial. BMJ Open 2023;13:e073709. © The Author(s) 2023. Published by BMJ. This article is distributed under the terms of the Creative Commons Attribution (CC BY 4.0) licence.
