# Supplementary material for: A multidomain, nurse-led, mobile health-supported intervention for dementia risk reduction in primary care (HAPPI MIND): a pragmatic, single-blind, two-arm, cluster randomised controlled trial
Source: Lancet Reg Health West Pac. 2026 Oct 20;75:102000. doi: 10.1016/j.lanwpc.2026.102000 (PMC13717166; doi:10.1016/j.lanwpc.2026.102000)
Supplement: Supplementary file 3 [file mmc2.docx]

# **Supplementary Table 1: Modifiable risk factors, definitions, and methods of assessment**

| **Risk factor** | **Definition of risk factor** | **Method of assessment** |
| --- | --- | --- |
| Hypertension | ≥140 mm Hg systolic and/or ≥90 mm Hg diastolic | Measured by a health professional on more than two occasions in the past 12 months |
| Hyperlipidaemia | >4.0 mmol/L total cholesterol; <1.0 mmol/L HDL-C; >2.0 mmol/L LDL-C; >2.5 mmol/L non-HDL-C; >2.0 mmol/L triglycerides | Laboratory blood test (fasting) at least once in the past 12 months |
| Type 2 Diabetes | HbA1c >6.5%; fasting glucose ≥7.0 mmol/L; random glucose ≥11.1 mmol/L | Laboratory blood test at least once in the past 12 months |
| Obesity | BMI ≥30 kg/m² | Self-reported or measured by health professional (height and weight) |
| Smoking | Current smoker | Self-report |
| Physical Inactivity | Exercise <30 min/day, <3 times/week | Self-report |
| Poor Diet | MIND diet score <7.5 | Self-report |
| Excessive Alcohol Use | >17 standard drinks/week | Self-report |
| Depression | Centre for Epidemiologic Studies Depression (CESD-10) scale ≥10 score | Health professional assessed or self-report |
| Social Isolation | Social isolation | Answered "yes" to ≥2 of: lives alone, no weekly contact with family/friends, no weekly social activities, or self-reported loneliness |
| Lack of Cognitive Stimulation | Lack of mentally stimulating activities | Health professional assessed or self-report: fewer than half the days in the past 2 weeks doing cognitive activities (e.g., reading, puzzles, music) |
| Current Hearing Impairment | Suspected or poorly managed hearing impairment | Self-report: "Yes" to trouble hearing on more than half the days in the past 2 weeks |
| Family History of Dementia | Parent or sibling diagnosed with dementia | Self-report |
| Low Education | Less than 8 years of schooling | Self-report |

**Note:** A standard drink in Australia contains 10 grams (12.5 mL) of pure alcohol. **MIND**: Mediterranean-DASH Intervention for Neurodegenerative Delay.

**Supplementary Table 2: Prevalence of dementia risk factors by study group in middle-aged Australian adults**

| **Risk factor** | **Intervention, n = 201** | **Control, n = 202** | **Total, n = 403** |
| --- | --- | --- | --- |
| Uncontrolled hypertension* | 97 (50.3) | 109 (44.9) | 206 (52.7) |
| Uncontrolled diabetes^†^ | **49 (25.8)** | **30 (16.2)** | 79 (21.1) |
| Uncontrolled hyperlipidaemia^‡^ | 173 (91.5) | 173 (93.5) | 346 (92.5) |
| Depression | 77 (38.3) | 87 (43.1) | 164 (40.7) |
| Physical inactivity | **91 (45.3)** | **62 (30.7)** | 153 (38.0) |
| Social isolation | 30 (14.9) | 40 (19.8) | 70 (17.4) |
| Current smoking | 38 (18.9) | 38 (18.8) | 76 (18.9) |
| Excessive alcohol intake | 14 (7.0) | 17 (8.4) | 31 (7.7) |
| Low cognitive activity | 149 (74.1) | 143 (70.8) | 292 (72.5) |
| Hearing impairment^§^ | 23 (11.4) | 20 (10.0) | 43 (10.7) |
| Family history of dementia^\|\|^ | 68 (34.2) | 65 (32.7) | 133 (33.4) |
| Obesity | 124 (61.7) | 108 (53.5) | 232 (57.6) |
| Poor diet | 53 (26.4) | 45 (22.3) | 98 (24.3) |
| Low level of education | 4 (2.0) | 1 (0.5) | 5 (1.2) |

*Data were missing for 12 participants; †Data were missing for 28 participants; ‡Data were missing for 29 participants; §Data were missing for 02 participants; ||Data were missing for 05 participants.

Bold indicates statistical significance (p<0.05).

**Supplementary Table 3: Distribution of individual ANU-ADRI components at baseline and 12 months by treatment group**

| **ANU-ADRI component** | **Category*** | **Baseline n (%)** | | **12 months n (%)** | |
| --- | --- | --- | --- | --- | --- |
|  |  | **Intervention (n=201)** | **Control (n=202)** | **Intervention (n=200)** | **Control (n=201)** |
| **Protective factors** | | | | | |
| Physical activity | Low | 91 (45.3) | 62 (30.7) | 54 (27.0) | 59 (29.4) |
|  | Medium | 64 (31.8) | 79 (39.1) | 67 (33.5) | 73 (36.3) |
|  | High | 46 (22.9) | 61 (30.2) | 80 (40.0) | 69 (34.3) |
| Cognitive activity | Low | 149 (74.1) | 143 (70.8) | 111 (55.5) | 119 (59.2) |
|  | Medium | 50 (24.9) | 58 (28.7) | 79 (39.5) | 77 (38.3) |
|  | High | 2 (1.0) | 1 (0.5) | 10 (5.0) | 5 (2.5) |
| Fish intake | Low | 106 (52.7) | 91 (45.0) | 69 (34.5) | 76 (37.8) |
|  | Low–moderate | 89 (44.3) | 100 (49.5) | 97 (48.5) | 108 (53.7) |
|  | Moderate | 6 (3.0) | 9 (4.5) | 9 (4.5) | 11 (5.5) |
|  | High | 0 (0.0) | 2 (1.0) | 24 (12.0) | 7 (3.5) |
| **Mixed factor** | | | | | |
| Alcohol intake | None | 35 (17.4) | 37 (18.3) | 36 (18.0) | 40 (19.9) |
|  | Moderate/High | 152 (75.6) | 148 (73.3) | 150 (75.0) | 143 (71.1) |
|  | Excessive | 14 (7.0) | 17 (8.4) | 14 (7.0) | 18 (9.0) |
| **Risk factors** | | | | | |
| High cholesterol | No | 139 (69.2) | 147 (72.8) | 140 (70.0) | 144 (71.6) |
|  | Yes | 62 (30.8) | 55 (27.2) | 60 (30.0) | 57 (28.4) |
| Diabetes | No | 132 (65.7) | 150 (74.3) | 117 (58.5) | 134 (66.7) |
|  | Yes | 69 (34.3) | 52 (25.7) | 84 (42.0) | 67 (33.3) |
| Body mass index | Normal | 88 (43.8) | 98 (48.5) | 108 (54.0) | 108 (53.7) |
|  | Overweight | 34 (16.9) | 37 (18.3) | 32 (16.0) | 29 (14.4) |
|  | Obese | 79 (39.3) | 67 (33.2) | 59 (29.5) | 64 (31.8) |
| Smoking | Never | 73(36.3) | 78(38.6) | 72 (36.0) | 77 (38.3) |
|  | Former smoker | 90(44.8) | 86(42.6) | 92 (46.0) | 86 (42.8) |
|  | Current smoker | 38(18.9) | 38(18.8) | 36 (18.0) | 38 (18.9) |
| Depression | No | 124 (61.7) | 115 (56.9) | 135 (67.5) | 131 (65.2) |
|  | Yes | 77 (38.3) | 87 (43.1) | 65 (32.5) | 70 (34.8) |
| Social engagement | High | 104 (51.7) | 95 (47.0) | 86 (43.0) | 90 (44.8) |
|  | Medium–high | 67 (33.3) | 67 (33.2) | 75 (37.5) | 64 (31.8) |
|  | Low–medium | 22 (10.9) | 30 (14.9) | 14 (7.0) | 31 (15.4) |
|  | Low | 8 (4.0) | 10 (5.0) | 25 (12.5) | 16 (8.0) |
| Education | High | 140 (69.7) | 149 (73.8) | 139 (69.5) | 149 (74.1) |
|  | Medium | 57 (28.4) | 52 (25.7) | 57 (28.5) | 51 (25.4) |
|  | Low | 4 (2.0) | 1 (0.5) | 4 (2.0) | 1 (0.5) |
| Traumatic brain injury | No | 188(93.5) | 185(91.6) | 159 (79.5) | 170 (84.6) |
|  | Yes | 13(6.5) | 17(8.4) | 41 (20.5) | 31 (15.4) |
| Occupational pesticide exposure | No | 168(83.6) | 177(87.6) | 156 (78.0) | 168 (83.6) |
|  | Yes | 33(16.4) | 25(12.4) | 44 (22.0) | 33 (16.4) |

* Categories correspond to the original ANU-ADRI scoring algorithm. The ANU-ADRI scoring system is provided in Supplementary File 2. Twelve-month values are based on pooled estimates from 20 multiply imputed datasets; pooled frequencies were rounded to the nearest whole number.

**Supplementary Table 4: Adjusted within-group changes and between-group differences in continuous dementia risk factor clinical and biomarker measures from baseline to 12 months (MD, 95% CI, *P-value*).**

| **Variable** | **Within-group difference** | | **Between-group difference** | ***% Showing improvement**** |
| --- | --- | --- | --- | --- |
|  | ***Intervention (n=200)*** | ***Control (n=201)*** |  |  |
| Low-density lipoprotein (LDL) | **0.264 (0.035 to 0.493)** | **0.212 (0.000 to 0.423)** | 0.052 (−0.123 to 0.227), 0.560 | 54.6 / 56.1 |
| Participants with baseline LDL >2.0 mmol/L | **0.317 (0.060 to 0.575)** | **0.281 (0.046 to 0.516)** | 0.036 (-0.155 to 0.228), 0.708 | 57.5 / 57.6 |
| Total cholesterol^†^ | 0.134 (−0.143 to 0.411) | 0.063 (−0.185 to 0.311) | 0.071 (−0.155 to 0.296), 0.536 | 53.1 / 51.8 |
| Participants with baseline total cholesterol >4.0 mmol/L | 0.212 (−0.101 to 0.525) | 0.149 (−0.141 to 0.439) | 0.063 (−0.157 to 0.283), 0.574 | 57.0 / 54.8 |
| Participants with baseline total cholesterol >6.5 mmol/L | **1.935 (0.965 to 2.906)** | **2.255 (1.202 to 3.308)** | −0.320 (−0.972 to 0.332), 0.335 | 73.5 / 87.8 |
| High-density lipoprotein (HDL) | −0.005 (−0.115 to 0.105) | 0.066 (−0.042 to 0.173) | −0.071 (−0.165 to 0.023), 0.138 | 43.9 / 49.0 |
| Systolic blood pressure (SBP)^†^ | −1.745 (−4.862 to 1.372) | 0.160 (−2.907 to 3.228) | −1.906 (−4.605 to 0.793), 0.166 | 46.0 / 49.9 |
| Participants with baseline SBP ≥140 mm Hg | 3.980 (−1.716 to 9.676) | **8.451 (3.210 to 13.691)** | −4.471 (−9.391 to 0.450), 0.075 | 57.0 / 67.8 |
| Diastolic blood pressure (DBP) | 0.596 (−1.718 to 2.910) | 1.613 (−0.631 to 3.856) | −1.017 (−3.223 to 1.190), 0.366 | 52.7 / 57.6 |
| Participants with baseline DBP ≥90 mm Hg | **4.969 (1.589 to 8.349)** | **5.744 (2.777 to 8.711)** | −0.775 (−3.815 to 2.265), 0.617 | 70.2 / 72.8 |
| CESD-10 | −0.066 (−1.229 to 1.097) | 0.274 (−0.811 to 1.358) | −0.340 (−1.285 to 0.606), 0.481 | 49.8 / 48.6 |
| Participants with baseline CESD ≥10 score | 0.434 (−1.977 to 2.846) | 1.918 (−0.274 to 4.109) | −1.483 (−3.145 to 0.179), 0.080 | 60.1 / 69.3 |
| Body mass index (BMI) | 0.639 (−0.705 to 1.984) | −0.221 (−1.028 to 0.585) | 0.861 (−0.138 to 1.860), 0.090 | 55.7 / 49.6 |
| Participants with baseline BMI ≥25 kg/m^2^ | 1.054 (−0.317 to 2.425) | 0.104 (−0.690 to 0.899) | 0.950 (−0.279 to 2.178), 0.127 | 60.6 / 51.1 |
| Participants with baseline BMI ≥30 kg/m^2^ | 1.436 (−0.449 to 3.320) | 0.236 (−0.792 to 1.264) | 1.200 (−0.553 to 2.952), 0.175 | 63.6 / 50.9 |
| Fasting blood glucose^†^ | 0.223 (−0.229 to 0.676) | −0.019 (−0.524 to 0.487) | 0.242 (−0.167 to 0.650), 0.244 | 49.1 / 47.3 |
| Participants with baseline blood glucose ≥7.0 mmol/L | 1.644 (0.243 to 3.046) | 1.098 (−0.322 to 2.519) | 0.546 (−0.786 to 1.878), 0.421 | 67.3 / 64.1 |
| Haemoglobin A1c (HbA1c) | −0.071 (−0.323 to 0.182) | −0.147 (−0.396 to 0.101) | 0.077 (−0.164 to 0.317), 0.527 | 51.7 / 48.8 |
| Participants with baseline HbA1c >6.5% | −0.185 (−0.897 to 0.526) | −0.260 (−1.030 to 0.510) | 0.075 (−0.743 to 0.892), 0.858 | 53.3 / 61.9 |

**Note:** Deceased participants (n=2) were excluded from the analyses. Rows labelled according to baseline clinical thresholds represent analyses of the continuous outcome restricted to participants who met the specified baseline criterion; the reported mean differences do not represent differences in proportions. Adjusted estimates were obtained using linear mixed-effects models accounting for clustering by clinic and adjusting for prespecified baseline covariates. Analyses were based on multiply imputed datasets, with parameter estimates pooled using Rubin's rules. No adjustment for multiple testing was applied. Bold text indicates statistically significant results (p<0·05).

Percentage of participants whose clinical or biomarker measure improved from baseline to 12 months. Where appropriate, change scores were transformed so that positive values consistently indicated improvement; accordingly, positive between-group MDs favour the intervention group and negative MDs favour the control group.

† Clinical outcomes (lipid, blood pressure, and glucose measures) were not adjusted for concurrent use or changes in relevant medications (e.g., lipid-lowering, antihypertensive, or antidiabetic therapies) during follow-up.

**BMI**: body mass index; **CESD-10:** Center for Epidemiologic Studies Depression Scale-10; **DBP**: diastolic blood pressure; **HbA1c**: haemoglobin A1c; **HDL**: high-density lipoprotein; **LDL**: low-density lipoprotein; **MD**: mean difference; **SBP**: systolic blood pressure.

**Supplementary Table 5: Change in the ANU-ADRI total score by risk factor targeted status (MD, 95%CI, P-value; Intervention group only - n = 200)**

| **Risk factor** | **N Targeted** | **Targeted** | **Not Targeted** | **Between-group difference** |
| --- | --- | --- | --- | --- |
| High cholesterol | 41 | 1.803 (−0.716 to 4.321) | 1.287 (−0.818 to 3.391) | 0.516 (−1.499 to 2.531), 0.616 |
| Hypertension | 15 | 2.492 (−0.977 to 5.961) | 1.358 (−0.672 to 3.389) | 1.134 (−1.944 to 4.212), 0.470 |
| Diabetes / High blood sugar | 22 | 0.457 (−2.383 to 3.297) | 1.399 (−0.685 to 3.483) | −0.942 (−3.603 to 1.719), 0.487 |
| Overweight/Obesity | 143 | 1.502 (−0.508 to 3.513) | 0.811 (−1.884 to 3.506) | 0.691 (−1.073 to 2.456), 0.442 |
| Physical inactivity | 160 | 1.202 (−0.713 to 3.117) | 1.866 (−1.387 to 5.119) | −0.664 (−3.098 to 1.771), 0.590 |
| Low cognitive activity | 138 | **1.975 (0.045 to 3.905)** | 0.078 (−2.667 to 2.824) | 1.897 (−0.025 to 3.819), 0.053 |
| Sleep problem | 16 | 1.565 (−1.392 to 4.522) | 1.328 (−0.839 to 3.494) | 0.237 (−2.755 to 3.230), 0.876 |
| Unhealthy / Poor diet | 138 | 1.461 (−0.428 to 3.350) | 1.138 (−1.848 to 4.123) | 0.324 (−1.878 to 2.525), 0.771 |
| Low fish intake | 70 | 1.611 (−0.568 to 3.791) | 1.277 (−0.916 to 3.470) | 0.334 (−1.449 to 2.117), 0.713 |
| Smoking | 14 | 0.492 (−2.840 to 3.824) | 1.475 (−0.601 to 3.551) | −0.983 (−3.946 to 1.980), 0.516 |
| Alcohol use | 32 | **0.981 (−1.693 to 3.655)** | 1.417 (−0.664 to 3.498) | −0.436 (−2.516 to 1.644), 0.681 |
| Social isolation | 15 | **4.675 (1.519 to 7.832)** | 1.039 (−1.037 to 3.114) | **3.637 (0.782 to 6.491), 0.013** |
| Depression | 17 | **2.945 (−0.229 to 6.120)** | 1.258 (−0.808 to 3.325) | 1.687 (−1.057 to 4.432), 0.228 |
| Stress | 5 | 0.214 (−4.836 to 5.265) | 1.378 (−0.681 to 3.437) | −1.164 (−5.995 to 3.668), 0.637 |
